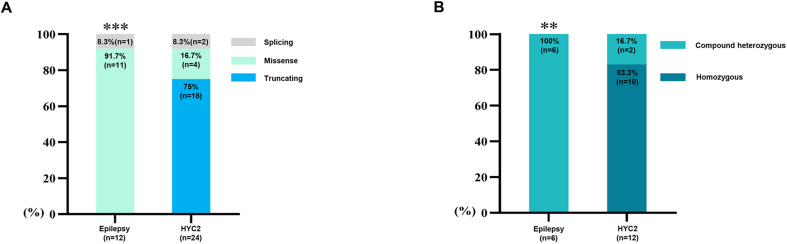# *MPDZ* variants associated with epilepsies and/or febrile seizures and the individualized genotype-phenotype correlation

**DOI:** 10.1016/j.gendis.2023.06.006

**Published:** 2023-07-13

**Authors:** Junxia Luo, Yun Li, Yong Lv, Xin Li, Bing Qin, Chuanfang Cheng, Xiaorong Liu, Weiping Liao, Jie Wang, Zaifen Gao

**Affiliations:** a Department of Epilepsy Center, Children's Hospital Affiliated to Shandong University, Jinan, Shandong 250022, China; b Department of Epilepsy Center, Jinan Children's Hospital, Jinan, Shandong 250022, China; c Department of Brain Function and Neuroelectrophysiology, The Affiliated Nanhua Hospital, Hengyang Medical School, University of South China, Hengyang, Hunan 421001, China; d Department of Pediatrics, The First Affiliated Hospital of University of Science and Technology of China (Anhui Provincial Hospital), Hefei, Anhui 230001, China; e Department of Pediatrics, The Second Hospital, Cheeloo College of Medicine, Shandong University, Jinan, Shandong 250033, China; f Epilepsy Center and Department of Neurosurgery, The First Affiliated Hospital of Jinan University, Guangzhou, Guangdong 510630, China; g Department of Cardiology, The Second Affiliated Hospital of Guangzhou Medical University, Guangzhou, Guangdong 510260, China; h Institute of Neuroscience and Department of Neurology of the Second Affiliated Hospital of Guangzhou Medical University, Guangzhou, Guangdong 510260, China; i Key Laboratory of Neurogenetics and Channelopathies of Guangdong Province and the Ministry of Education of China, Guangzhou, Guangdong 510260, China

The multiple PDZ domain crumbs cell polarity complex component gene (*MPDZ*; MIM: 603785), is highly expressed in the brain across the whole lifespan. It encodes the multiple PDZ domain protein, which is a member of the NMDAR signaling complex that may play a role in the control of AMPAR potentiation and synaptic plasticity in excitatory synapses.^1^ Previously, *MPDZ* variants have been demonstrated to be associated with autosomal recessive congenital hydrocephalus-2 (HYC2; MIM: 615219) which is commonly complicated by brain abnormalities and developmental delay. Seizures were reported in only one case. The association between *MPDZ* and epilepsy requires clarification.

In the present study, we performed trio-based whole-exome sequencing in a cohort of 168 epilepsy cases without acquired causes (supplementary materials and methods). *MPDZ* variants were identified in six unrelated families with epilepsy (Fig. 1A–C and Table S1). None of the affected individuals with *MPDZ* variants had pathogenic or likely pathogenic variants in the genes known to be associated with epileptic phenotypes.^2^ All variants present no or extremely low allele frequencies in the gnomAD database and statistically higher frequency in this cohort than in populations of gnomAD (Table S2). The recessive variants burden analysis also showed a statistically significant difference of *MPDZ* variants in this cohort compared with the expected number by chance in the controls of all populations in the Exome Aggregation Consortium (*P* = 9.198 × 10^−8^).

**Figure 1:**
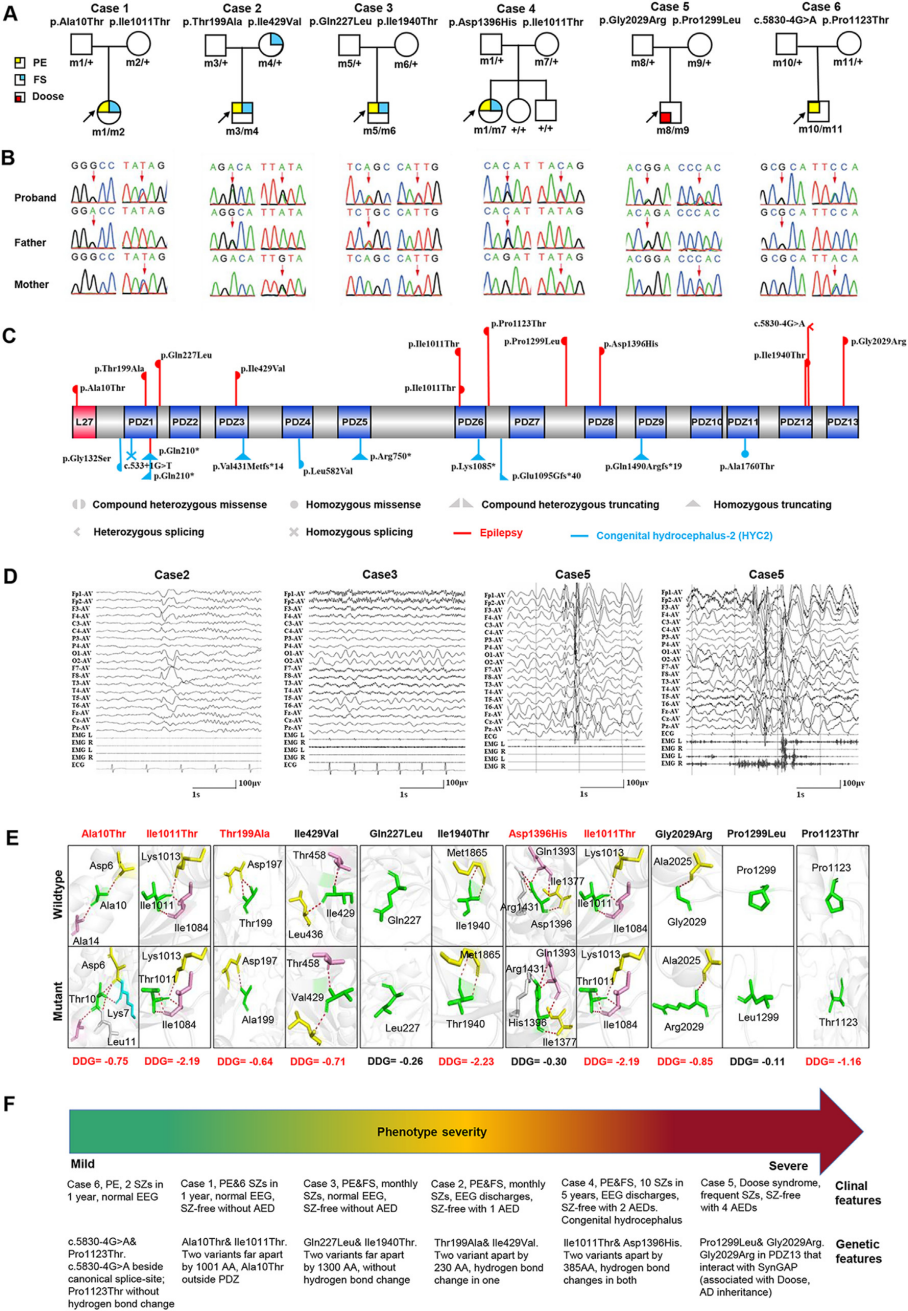
Genetic and clinical data of cases with *MPDZ* variants, and genotype-phenotype correlation in each case. **(A)** Pedigrees of the cases with *MPDZ* mutations and their corresponding phenotypes. Individuals with heterozygous mutation are indicated by m/+, those with compound heterozygous mutation are indicated by m/m, and those negative for mutation are indicated by +/+. The probands are indicated by black arrows. The phenotype of each case is indicated by different symbols in the figure. **(B)** DNA sequencing chromatograms of the cases with *MPDZ* mutations. The positions of the mutations are indicated by red arrows. **(C)** Schematic illustration of the MPDZ protein and the location of the *MPDZ* mutations identified in this study and congenital hydrocephalus-2 (HYC2). Variants at the same level indicate the pair of compound heterozygous. **(D)** Electroencephalography data of the cases with *MPDZ* mutations. Interictal EEG in case 2 showed right frontotemporal spike-slow waves; interictal EEG in case 3 showed normal EEG; interictal EEG in case 5 showed generalized spikes, spike-slow waves, polyspike-slow waves, and slow waves; ictal EEG in case 5 showed myoclonic-atonic seizures. **(E)** Hydrogen bond changes and DDG value of *MPDZ* missense mutations identified in this study. **(F)** Epileptic spectrum of *MPDZ* variants and genotype-phenotype characteristics in each case. AA, amino acid; AD, autosomal dominant; AED, anti-epileptic medication; Doose, Doose syndrome (epilepsy with myoclonic-atonic seizures); EEG: electroencephalogram; FS, febrile seizures; PE, partial epilepsy; SZ, seizure.

The detailed clinical manifestations were summarized in Table S1. The median seizure-onset age of the probands was two years old. Five cases were diagnosed as partial (focal) epilepsy, among which four probands and a parent had febrile seizures. The electroencephalogram (EEG) recordings of two cases with partial epilepsy showed focal abnormalities with features of idiopathic partial epilepsies, including shifting, bilateral, or multiple focal discharges with normal backgrounds; while the other three cases with partial epilepsy showed normal EEG recordings. One case was diagnosed with Doose syndrome, in whom ictal myoclonic-atonic and myoclonic seizures and interictal generalized epileptic discharges in EEGs were recorded (Fig. 1D). Congenital hydrocephalus has been observed in case 4 at the age of seven months. Brain magnetic resonance imaging was normal in the other five cases. All patients had normal intelligence and development. Two cases were seizure-free without anti-epileptic therapy; the other four cases became seizure-free for at least one year on monotherapy or in combination treatment (Table S1).

The missense variants were predicted to be damaging and conserved by *in silico* tools (Table S3). The molecular effect of the missense variants was further analyzed by using AlphaFold (https://alphafold.ebi.ac.uk/) and PyMOL 2.7. Two pairs of compound heterozygous variants had hydrogen bond changes in both bi-allele variants. One pair of compound heterozygous variants had hydrogen bond changes in one of the paired variants. The remaining six variants did not show a significant effect on hydrogen bonding, but four of which were predicted to largely decrease the protein stability (Fig. 1E).

The *MPDZ* missense variants identified in this study are scattered over the whole MPDZ protein and mostly assemblies located at or near the PDZ domains (Fig. 1C). It was noticed that two cases (case 1 and case 3) with compound heterozygous variants with two variants located far apart from each other (a distance away of 1001 and 1300 amino acids, respectively), are associated with milder epileptic phenotypes and became seizure-free without anti-epileptic therapy. They presented normal EEG recordings (Fig. 1D). Case 4 carried two variants that were located close to each other and in PDZ domains, and had hydrogen bond changes in both bi-allele variants, in whom congenital hydrocephalus was observed.

Case 2 harbored variant Ile429Val that originated from his mother with febrile seizures and located in PDZ3 of MPDZ, which interacts with DAPLE (encoded by *CCDC88C*) that is potentially associated with neurodegenerative diseases, congenital, hydrocephalus, and epilepsy in an autosomal dominant/recessive inheritance.^3^ The patient diagnosed with Doose syndrome (case 5) had the variant located in PDZ13 of MPDZ (Gly2029Arg), which interacts with SynGAP (encoded by *SYNGAP1*) that is associated with epileptic encephalopathy.^1^^,^^4^

The genotype-phenotype characteristics of each case were summarized in Figure 1F. The molecular sub-regional effect, including the distance between the two variants of the paired compound heterozygous variants, hydrogen bond changes, mutation types, and downstream interacting proteins of MPDZ, explained the phenotypic heterogeneity in each case.

Previously, *MPDZ* variants have been reported in 12 cases with HCY2 (Fig. 1C and Table S4). The HCY2-associated *MPDZ* variants were homozygous truncating variants mostly, whereas the variants related to epilepsy (from this study) were mostly compound heterozygous missense variants with statistically significant differences (Fig. S1).

HCY2-associated *MPDZ* variants were mostly truncating variants, suggesting that the loss of function of *MPDZ* may be a potentially pathogenic mechanism. Previously, the bi-allele truncating variants were associated with a more severe phenotype of HCY2; while the few cases with bi-allele missense variants were associated with a milder phenotype. The *MPDZ* variants identified in the present study were missense variants mostly, and variant c.5830-4G > A (beside the canonical splice site) was potentially associated with mild damage. All the patients presented normal development, good responses to anti-epileptic drugs, and eventually became seizure-free. These findings suggest that the severe functional impairment of *MPDZ* variants was associated with severe phenotypes of HCY2, whereas milder functional alterations were potentially associated with relatively mild phenotypes of epilepsy with a favorable outcome.

Previously, the two bi-missense mutations associated with HYC2 are homozygous missense or compound heterozygous missense mutations with each variant located close (450 amino acids away). In the present study, case 4 with variants located close to each other (Ile1011Thr/Asp1396His, 385 amino acids away) and altered hydrogen bonding in both also had hydrocephalus. In contrast, cases 1 and 3 with compound heterozygous missense mutations that were located relatively far away (a distance of 1001 and 1300 amino acids, respectively) are associated with a milder epileptic phenotype with normal EEG findings and favorable outcome without anti-epileptic therapy. These findings suggested that the distance between the two variants of the paired compound heterozygous variants was potentially associated with the degree of damage and subsequently the severity of phenotype.

The *MPDZ* variants identified in this study are mostly assemblies located at or near the PDZ domains. Previous studies have shown that several PDZ domains and their downstream interacting proteins were associated with epilepsy. PDZ10 of MPDZ interacts with the 5-hydroxytryptamine type 2C receptors (HTR2C), which presented epilepsy phenotypes in knockout mice model.^5^ PDZ3 of MPDZ interacts with DAPLE (encoded by *CCDC88C*) that is associated with neurodegenerative diseases, congenital hydrocephalus, and epilepsy in an autosomal dominant/recessive inheritance,^3^ potentially explaining the heterozygous variants associated with febrile seizures. PDZ13 of MPDZ combines with SynGAP encoded by *SYNGAP1*, playing a critical role in synaptic NMDAR-dependent AMPA receptor trafficking.^4^ *SYNGAP1* variants have been frequently reported to be associated with epileptic encephalopathy, including Doose syndrome in an autosomal dominant inheritance. In the present study, case 5 harboring variant Gly2029Arg that is located in the PDZ13 domain, was also diagnosed with Doose syndrome with frequent myoclonic-atonic and myoclonic seizures with general polyspike-slow waves in EEG. These findings provided further evidence in supporting the association between *MPDZ* and epilepsy and emphasized the effect of molecular sub-regional of *MPDZ* variants.

In conclusion, this study suggests *MPDZ* variants are potentially associated with epilepsy and/or febrile seizures. The genotype and molecular sub-regional effect of *MPDZ* help explain the phenotypic heterogeneity and provide critical information for individualized precise medicine.

## Ethics declaration

All procedures performed were in accordance with the ethical standards of the institutional committee. The present study was approved by the Ethics Committee of Children's Hospital Affiliated to Shandong University (Jinan Children's Hospital)

## Author contributions

Z.G. and J.W. designed the study. Y.L., J.L., Y.L., X.L., and B.Q. completed the recruitment of the patients and the analysis of the clinical data. J.L., X.L., and J.W. completed the analysis of the genetic data. Y.L. and C.C. prepared the figures. J.L. and J.W. wrote the manuscript. W.L. revised the manuscript. All authors read and approved the final manuscript.

## Conflict of interests

The authors stated that they had no interests which might be perceived as posing a conflict or bias.

## Funding

This work was funded by the 10.13039/501100001809National Natural Science Foundation of China (No. 82201609), Shandong Medical and Health Science and Technology Development Plan (China) (No. 202106010271), Scientific Research Project of Hunan Provincial Health Commission (China) (No. D202303077290), Guangdong Basic and Applied Basic Research Foundation (China) (No. 2021A1515111064), and Science and Technology Project of Guangzhou, Guangdong, China (No. 202102021059, 202201020106, 202235395).

## Figures and Tables

**figs1:**