# Supplementary material for: MPDZ variants associated with epilepsies and/or febrile seizures and the individualized genotype-phenotype correlation
Source: Genes Dis. 2023 Jul 13;11(3):101032. doi: 10.1016/j.gendis.2023.06.006 (PMC10825275; doi:10.1016/j.gendis.2023.06.006)
Supplement: Multimedia component 1 [file mmc1.docx]

**Supplementary Data**

1. **Supplementary Materials and Methods**

**Subjects**

We totally recruited 168 unrelated cases (trios) with epilepsy for genetic screening during 2018 to 2022 from five hospitals, including The Children's Hospital Affiliated to Shandong University (Jinan Children's Hospital), The Affiliated Nanhua Hospital of University of South China, The First Affiliated Hospital of University of Science and Technology of China (Anhui Provincial Hospital), The Second Hospital of Shandong University, and The First Affiliated Hospital of Jinan University. We comprehensively collected the clinical information of the patients, including the age of seizure onset, seizure type and frequency, family history of epilepsy and febrile seizures, general and neurological examination results, response to anti-seizure medications, and brain magnetic resonance imaging (MRI) scans. All of the patients were evaluated by long-term video-electroencephalogram (video-EEG) monitoring that included open-close eyes test, hyperventilation, intermittent rhythmic photic stimulation, and sleeping recording. The EEG results were reviewed by two qualified electroencephalographers to ensure data quality and consistency. Epileptic seizures and epilepsy syndromes were diagnosed according to the criteria of the Commission on Classification and Terminology of the International League Against Epilepsy (1981, 1989, 2010, and 2017).^1-4^ All enrolled subjects were diagnosed as epilepsy that was characterized by focal or generalized seizures. Generalized epilepsies were diagnosed based on a range of seizure types including absence, spasms, myoclonic, clonic, atonic, tonic, and tonic-clonic seizures, supported by the findings of typically generalized discharges on EEG or normal EEG. Focal epilepsies were used to denote cases with partial seizures and/or unifocal/multifocal epileptiform discharges on EEG. Patients with acquired etiologies, such as tumor, trauma, infection, immunity, and stroke, were excluded. All of the subjects were followed up for at least one year.

This study adhered to the guidelines of the International Committee of Medical Journal Editors with regard to patient consent for research or participation, and approved by the ethics committee of Children's Hospital Affiliated to Shandong University (Jinan Children's Hospital). Written informed consents were obtained from the patients and their legal guardians.

**Whole-exome sequencing and genetic analysis**

The blood samples were collected from the probands, their parents, and other available family members to determine the origin of the identified genetic variants. The genomic DNAs from the blood samples were extracted by using the Qiagen Flexi Gene DNA kit (Qiagen, Hilden, Germany). Trio-based WES was performed with NextSeq500 sequencing instruments (Illumina, San Diego, California) according to the standard procedures as previously described.^5-7^ We adopted a case-by-case analytical approach to identify candidate causative variants in each trio. Firstly, we prioritized the rare variants with a minor allele frequency <0.005 in the Genome Aggregation Database (gnomAD) (http://gnomad.broadinstitute.org/). We retained potentially pathogenic variants, including frameshift, nonsense, canonical splice site, initiation codon, and missense variants predicted as being damaging by in silico tools (http://varcards.biols.ac.cn/). Finally, we analyzed the potential disease-causing mutations in each case under the following five models as we recently reported ^8^: 1) Epilepsy-associated gene model; 2) De novo mutation dominant model; 3) Autosomal recessive inheritance model, including homozygous and compound heterozygous variants; 4) X-linked model; 5) Co-segregation analysis model, if necessary. Genes with repetitively identified de novo variants, bi-allelic variants, hemizygous variants, or variants with segregations, were selected for further studies to define the gene-disease association. *MPDZ* emerged as one of the candidate genes with recurrent bi-allelic variants in this cohort. The other potential candidate genes were not the targets of this study. All of the candidate pathogenic variants were validated by Sanger sequencing. Conservation of the mutated positions was evaluated by generating multiple sequence alignments of different species. The variants in *MPDZ* gene in this study were all annotated to reference to the transcript NM_001261406.

**Mutation analysis and genotype-phenotype association analysis**

Protein modeling was performed to predict the effects of missense variants on molecular structure by using AlphaFold (https://alphafold.ebi.ac.uk/). The protein modeling (O75970, MPDZ_HUMAN) was directly downloaded from the website. PyMOL 2.7 was used for three-dimensional protein structure visualization and analysis. In additionally, we applied I-Mutant 3.0 program to predict the effect of *MPDZ* missense variants on protein stability, which was indicated by free energy change (DDG) (http://gpcr.biocomp.unibo.it/cgi/predictors/I-Mutant3.0/I-Mutant3.0.cgi). Variants were divided into three classes: large increase of protein stability (DDG > 0.5 kcal/mol), large decrease of protein stability (DDG < -0.5 kcal/mol), and neutral stability (-0.5 kcal/mol ≤ DDG ≤ 0.5 kcal/mol).

To evaluate the genotype-phenotype correlation, we reviewed all the relevant literatures about *MPDZ* variants reported previously, as well as the related phenotypes of the variants. All the variants were retrieved from the profession edition of Human Gene Variant Database (http://www.hgmd.cf.ac.uk/ac/index.php) and PubMed database (https://pubmed.ncbi.nlm.nih.gov/) up to December 2022.

**Statistical analysis**

R statistical software (v3.4.1) was used for statistical analyses. The frequency of the *MPDZ* variants in general populations and that in the cohort of partial epilepsy were analyzed by using a two-tailed Fisher’s exact test. The recessive *MPDZ* variants burden was also analyzed according to a recent research.^9^ A *p* value of < 0.05 was considered to be statistically significant.

**Reference:**

1. Commission on Classification and Terminology of the International League against Epilepsy. Proposal for revised clinical and electroencephalographic classification of epileptic seizures. *Epilepsia*. 1981;22(4):489-501.
2. Commission on Classification and Terminology of the International League against Epilepsy. Proposal for revised classification of epilepsies and epileptic syndromes. *Epilepsia*. 1989;30(4):389-399.
3. Berg AT, Berkovic SF, Brodie MJ, et al. Revised terminology and concepts for organization of seizures and epilepsies: report of the ILAE commission on classification and terminology, 2005–2009. *Epilepsia*. 2010;51(4):676-685.
4. Scheffer IE, Berkovic S, Capovilla G, et al. ILAE classification of the epilepsies: position paper of the ILAE commission for classificationand terminology. *Epilepsia*. 2017;58(4):512-521.
5. Wang JY, Zhou P, Wang J, et al. ARHGEF9 mutations in epileptic encephalopathy/intellectual disability: toward understanding the mechanism underlying phenotypic variation. *Neurogenetics*. 2018;19(1):9-16.
6. Cai K, Wang J, Eissman J, et al. A missense mutation in SLC6A1 associated with Lennox-Gastaut syndrome impairs GABA transporter 1 protein trafficking and function. *Experimental neurology*. 2019;320:112973.
7. Shi YW, Zhang Q, Cai K, et al. Synaptic clustering differences due to different GABRB3 mutations cause variable epilepsy syndromes. *Brain*. 2019;142(10):3028-44.
8. Wang J, Qiao JD, Liu XR, et al. UNC13B variants associated with partial epilepsy with favourable outcome [published correction appears in Brain. 2022 Apr 18;145(2):e5]. *Brain*. 2021;144(10):3050-3060.
9. Martin HC, Jones WD, McIntyre R, et al. Quantifying the contribution of recessive coding variation to developmental disorders. *Science*. 2018;362(6419):1161-1164.
