# Supplementary material for: MPDZ variants associated with epilepsies and/or febrile seizures and the individualized genotype-phenotype correlation
Source: Genes Dis. 2023 Jul 13;11(3):101032. doi: 10.1016/j.gendis.2023.06.006 (PMC10825275; doi:10.1016/j.gendis.2023.06.006)
Supplement: Multimedia component 2 [file mmc2.docx]

1. **Supplementary Tables**

**Table S1. Clinical features of the individuals with *MPDZ* variants**

| **Case**  **ID** | **Variants**  **(****NM_001261406)** | **Gender** | **Age** | **Seizure onset** | **Seizure course** | **Effective AEDs** | **Seizure-free duration** | **EEG** |  | **Brain**  **MRI** | **Development** | **Diagnosis** |
| --- | --- | --- | --- | --- | --- | --- | --- | --- | --- | --- | --- | --- |
| 1 | c.28C>T/p.Ala10Thr  c.3032A>G/p.Ile1011Thr | Female | 3 yr | 10 mo | sGTCS 5/yr in the first year, FS once at age 1yr | - | 1 yr | Normal |  | Normal | Normal | PE, FS |
| 2 | c.595A>G/p.Thr199Ala  c.1285A>G/p.Ile429Val | Male | 8 yr | 3 yr | FS 3-4/yr from age 3-5yr, sGTCS or CPS 1-2/mo at age 6yr | VPA | 2 yr | Bilateral occipital and temporal spike-slow and slow waves; Right frontotemporal spike-slow waves |  | Normal | Normal | PE, FS |
| 3 | c.680A>T/p.Gln227Leu  c.5819T>C/p.Ile1940Thr | Male | 4 yr | 8 mo | sGTCS 0-1/mo in the first year, FS 6 times at age 1yr | - | 2 yr | Normal |  | Normal | Normal | PE, FS |
| 4 | c.3032T>C/p.Ile1011Thr  c.4186G>C/p.Asp1396His | Female | 9 yr | 2 yr | FS 1-2/yr from age 2-5yr, sGTCS or CPS 4 times at age 7yr | LEV  LTG | 2 yr | Left centroparietal spike-slow waves during sleep |  | Mildly enlarged ventricles | Normal | PE, FS |
| 5 | c.3896C>T/p.Pro1299Leu  c.6085G>A/p.Gly2029Arg | Male | 4 yr | 2 yr | Myoclonic and myoclonic-atonic seizures 3-4/d for 1 yr | VPA  TPM  CNZ  LTG | 1 yr | Interictal: generalized spikes, spike-slow waves, polyspike-slow waves, slow waves  Ictal: myoclonic seizures, myoclonic-atonic seizures |  | Normal | Normal | Doose |
| 6 | c.5830-4G>A  c.3367C>A/p.Pro1123Thr | Male | 15 yr | 13 yr | sGTCS twice at age 13yr | OXC | 2 yr | Normal |  | Normal | Normal | PE |

Abbreviations: AEDs, anti-epileptic medications; CNZ, clonazepam; CPS, complex partial seizure; d, day; Doose, Doose syndrome (epilepsy with myoclonic-atonic seizures); EEG, electroencephalogram; FS, febrile seizures; LEV, levetiracetam; LTG, lamotrigine；MRI, magnetic resonance imaging; mo, month; OXC, oxcarbazepin; PE, partial epilepsy; sGTCS, secondary generalized tonic-clonic seizure; TPM, topiramate; VPA, valproate; yr, year.

**Table S2. Analysis of the aggregate frequency of *MPDZ* variants identified in this study**

|  | **Allele Count/Number in this study** | **Allele Count/Number in the gnomAD-all populations** | **Allele Count/Number in the controls of gnomAD-all populations** | **Allele Count/Number in the gnomAD-East Asian population** | **Allele Count/Number in the controls of gnomAD-East Asian population** |
| --- | --- | --- | --- | --- | --- |
| **Identified** ***MPDZ* Mutations** |  |  |  |  |  |
| c.28G>A/p.Ala10Thr | 1/336 | 8/248312 (3.222×10^-5^) | 4/108322 (3.693×10^-5^) | 7/17976 (3.894×10^-4^) | 3/8638 (3.473×10^-4^) |
| c.3032T>C/p.Ile1011Thr | 2/336 | 32/216566 (1.478×10^-4^) | 19/88550 (2.146×10^-4^) | 24/15148 (1.584×10^-3^) | 14/6538 (2.141×10^-3^) |
| c.595A>G/p.Thr199Ala | 1/336 | - | - | - | - |
| c.1285A>G/p.Ile429Val | 1/336 | 10/245614 (4.071×10^-5^) | 3/107346 (2.795×10^-5^) | 10/17802 (5.617×10^-4^) | 3/8574 (3.499×10^-4^) |
| c.680A>T/p.Gln227Leu | 1/336 | - | - | - | - |
| c.5819T>C/p.Ile1940Thr | 1/336 | 55/279704 (1.966×10^-4^) | 26/119066 (2.184×10^-4^) | 52/19492 (2.668×10^-3^) | 25/9536 (2.622×10^-3^) |
| c.4186G>C/p.Asp1396His | 1/336 | - | - | - | - |
| c.3896C>T/p.Pro1299Leu | 1/336 | 18/280156 (6.425×10^-5^) | 7/119384 (5.863×10^-5^) | 18/19528 (9.218×10^-4^) | 7/9548 (7.331×10^-4^) |
| c.6085G>A/p.Gly2029Arg | 1/336 | 286/278842 (1.026×10^-3^) | 119/118956 (1.0×10^-3^) | 0/19488 | 0/9542 |
| c.5830-4G>A  c.3367C>A/p.Pro1123Thr | 1/336  1/336 | 76/275362 (2.760×10^-4^)  - | 26/119066 (2.184×10^-4^)  - | 20/19238 (1.040×10^-3^)  - | 25/9536 (2.622×10^-3^) |
| **Total** | 12/336 (0.036) | 485/216566 (0.0022) | 204/88550 (0.0023) | 131/15148 (0.0086) | 77/6538 (0.012) |
| ***P* value** |  | 3.328×10^-11^ | 5.543×10^-11^ | 6.69×10^-5^ | 1.212×10^-3^ |
| **OR (95%CI)** |  | 16.500 (8.380-29.462) | 16.037 (8.071-29.014) | 4.245 (2.117-7.769) | 3.107 (1.523-5.817) |

*P* values and odds ratio were estimated with 2-sided Fisher’s exact test.

Abbreviations: CI, confidence interval; gnomAD, Genome Aggregation Database; OR, odd ratio

**Table S3. Bioinformatics Analysis of the *MPDZ* Variants**

| **Case** | **cDNA change (NM_001261406)** | **Protein change** | **Inheritance** | **MAF** | **MAF-EAS** | **SIFT** | **PP2_Var** | **Mutation-Taster** | **CADD** | **Fathmm-MKL** | **FitCons** | **GERP++** | | **PhastCons** |
| --- | --- | --- | --- | --- | --- | --- | --- | --- | --- | --- | --- | --- | --- | --- |
| Case 1 | c.28C>T c.3032A>G | p.Ala10Thr p.Ile1011T | Paternal Maternal | 3.222×10^-5^  1.478×10^-4^ | 3.894×10^-4^  1.584×10^-3^ | T (0.053)  D (0.006) | PD (0.999)  B (0.257) | DC (0.997)  DC (0.871) | D (29.4)  D (23.5) | D (0.994)  D (0.982) | D (0.707)  D (0.732) | C (5.68)  C (4.76) | | C (1.000)  C (1.000) |
| Case 2 | c.595A>G c.1285A>G | p.Thr199Ala p.Ile429Val | PaternalMaternal | -  4.071×10^-5^ | -  5.617×10^-4^ | T (0.194)  T (0.888) | B (0.246)  B (0.066) | DC (0.999)  DC (1.000) | D (22.7)  T (14.93) | D (0.985)  D (0.965) | D (0.706)  D (0.732) | C (6.07)  C (4.72) | C (1.000)  C (1.000) | |
| Case 3 | c.680A>T c.5732T>C | p.Gln227Leu p.Ile1940Thr | Paternal  Maternal | -  1.966×10^-4^ | -  2.668×10^-3^ | D (0.039)  D (0.001) | B (0.388)  PD (0.935) | P (0.879)  DC (1.000) | T (13.32)  D (31) | D (0.889)  D (0.992) | D (0.706)  D (0.732) | NC (-4.26)  C (5.52) | | C (1.000)  C (1.000) |
| Case 4 | c.3032T>C  c.4186G>C | p.Ile1011Thr  p.Asp1396His | Maternal  Paternal | 1.478×10^-4^  - | 1.584×10^-3^  - | D (0.006)  D (0.001) | B (0.257)  PD (1.0) | DC (0.871)  DC (1.000) | D (23.5)  D (33) | D (0.982)  D (0.990) | D (0.732)  D (0.732) | C (4.76)  C (5.72) | | C (1.000)  C (1.000) |
| Case 5 | c.3896C>T  c.5998G>A | p.Pro1299Leu  p.Gly2029Arg | Maternal  Paternal | 6.425×10^-5^  1.026×10^-3^ | 9.218×10^-4^  - | T (0.737)  D (0.002) | B (0.001)  PD (1.0) | P (1.000)  DC (1.0) | T (11.81)  D (35) | T (0.116)  D (0.981) | D (0.731)  D (0.732) | NC (-0.217)  C (5.52) | | NC (0.820)  C (1.000) |
| Case 6 | c.5830-4G>A c.3367C>A | p.Pro1123Thr | Paternal  Maternal | 2.760×10^-4^  - | 1.040×10^-3^  - | D (0.032) | PD (0.999) | DC (1.000) | D (23.8) | D (0.970) | D (0.706) | C (6.16) | | C (1.000) |

Abbreviations: B, benign; C, conserved; CADD, combined annotation dependent depletion; D, damaging; DC, disease-causing; Fathmm-MKL, functional analysis through hidden markov models–multiple kernels learning; FitCons, the fitness consequences of functional annotation; GERP, genomic evolutionary rate profiling; MAF, minor allele frequency from gnomAD; MAF-EAS, minor allele frequency from gnomAD-East Asian population; NC, non-conserved; P, polymorphism; PD, probably_damaging; PP2_Var, polyphen2_HVAR; SIFT, sorting intolerant from tolerant; T, tolerable.

**Table S4. Congenital hydrocephalus-related *MPDZ* mutations**

| Case ID | Nucleotide change | Amino acid change | Inheritance | Reference |
| --- | --- | --- | --- | --- |
| 1 | c.394G>A; c.1744C>G | p.Gly132Ser; p.Leu582Val | Maternal; Paternal | (1) |
| 2 | c.5278G>A | p.Ala1760Thr | Paternal; Maternal | (2) |
| 3 | c.533+1G>T | - | Paternal; Maternal | (3) |
| 4-7 | c.628C>T | p.Gln210Term | Paternal; Maternal | (2, 4-6) |
| 8 | c.628C>T; c.3283dup | p.Gln210Term; p.Glu1095Glyfs*40 | Maternal; Paternal | (6) |
| 9 | c.1291_1294del | p.Val431Metfs*14 | Paternal; Maternal | (3) |
| 10 | c.2248C>T | p.Arg750Term | Paternal; Maternal | (3) |
| 11 | c.3253A>T | p. Lys1085Term | Paternal; Maternal | (7) |
| 12 | c.4469delA | p.Gln1490Argfs*19 | Paternal; Maternal | (2) |

**References**

1. Al-Shamsi A, Hertecant JL, Souid AK, Al-Jasmi FA. Whole exome sequencing diagnosis of inborn errors of metabolism and other disorders in United Arab Emirates. Orphanet J Rare Dis. 2016;11(1):94.
2. Shaheen R, Sebai MA, Patel N, et al. The genetic landscape of familial congenital hydrocephalus. Ann Neurol. 2017;81(6):890-897.
3. Saugier-Veber P, Marguet F, Lecoquierre F, et al. Hydrocephalus due to multiple ependymal malformations is caused by mutations in the MPDZ gene. Acta Neuropathol Commun. 2017;5(1):36. Published 2017 May 1.
4. Al-Dosari MS, Al-Owain M, Tulbah M, et al. Mutation in MPDZ causes severe congenital hydrocephalus. J Med Genet. 2013;50(1):54-58.
5. Al-Hamed MH, Kurdi W, Khan R, et al. Prenatal exome sequencing and chromosomal microarray analysis in fetal structural anomalies in a highly consanguineous population reveals a propensity of ciliopathy genes causing multisystem phenotypes. Hum Genet.2022;141(1):101-126.
6. Jin SC, Dong W, Kundishora AJ, et al. Exome sequencing implicates genetic disruption of prenatal neuro-gliogenesis in sporadic congenital hydrocephalus. Nat Med. 2020;26(11):1754-1765.
7. Bertoli-Avella AM, Beetz C, Ameziane N, et al. Successful application of genome sequencing in a diagnostic setting: 1007 index cases from a clinically heterogeneous cohort. Eur J Hum Genet. 2021;29(1):141-153
